# Clinical detection and characterization of bacterial pathogens in the genomics era

**DOI:** 10.1186/s13073-014-0114-2

**Published:** 2014-11-29

**Authors:** Pierre-Edouard Fournier, Gregory Dubourg, Didier Raoult

**Affiliations:** Unité de Recherche sur les Maladies Infectieuses et Tropicales Emergentes, UM63, CNRS7278, IRD198, InsermU1095, Institut hospitalo-universitaire Méditerranée-Infection, Aix-Marseille University, Faculté de Medecine, 27 Blvd Jean Moulin, Marseille, 13385, cedex 5 France

## Abstract

The availability of genome sequences obtained using next-generation sequencing (NGS) has revolutionized the field of infectious diseases. Indeed, more than 38,000 bacterial and 5,000 viral genomes have been sequenced to date, including representatives of all significant human pathogens. These tremendous amounts of data have not only enabled advances in fundamental biology, helping to understand the pathogenesis of microorganisms and their genomic evolution, but have also had implications for clinical microbiology. Here, we first review the current achievements of genomics in the development of improved diagnostic tools, including those that are now available in the clinic, such as the design of PCR assays for the detection of microbial pathogens, virulence factors or antibiotic-resistance determinants, or the design of optimized culture media for ‘unculturable’ pathogens. We then review the applications of genomics to the investigation of outbreaks, either through the design of genotyping assays or the direct sequencing of the causative strains. Finally, we discuss how genomics might change clinical microbiology in the future.

**Electronic supplementary material:**

The online version of this article (doi:10.1186/s13073-014-0114-2) contains supplementary material, which is available to authorized users.

## The impact of next-generation sequencing in infectious disease diagnostics

Infectious diseases are one of the leading causes of human mortality worldwide [1]. Therefore, accurate diagnostic methods are required to optimize the clinical management of infected patients. However, the gold standard for the diagnosis of infectious diseases has long been the culture in growth-supporting media, including the isolation, identification and antibiotic-susceptibility testing of the causative microorganism. Currently, this diagnostic scheme takes a minimum of 24 hours. The introduction of the polymerase chain reaction (PCR) [2] method in the 1980s resulted in the development of a multitude of diagnostic tools that helped improve the efficiency of diagnostics and the characterization of infectious-disease agents by detecting and identifying their DNA. However, the design of these assays remained mostly empirical, being notably based on the use of the *16S* rRNA gene [3], until bacterial genome sequencing became a reality in the mid-1990s [4]. Microbial genomics, enabling a rational design of most molecular assays by selecting molecular targets according to their objective, has now had a major impact on the diagnosis and prevention of infectious diseases, with detection and identification of pathogens being directly performed within specimens without the need for culture [5].

Since 2005, the development of next-generation sequencing (NGS), together with decreasing costs for sequencers and reagents, has democratized genomics (Table 1) [6]. Currently, a bacterial genome sequence can be obtained within a few days for less than US$500 [6], and more than 38,000 genome sequences are available in public databases [7]. NGS has had many applications in medical microbiology, including the design of diagnostic and genotyping tools, the identification of virulence and antibiotic-resistance mechanisms and the development of specific culture media [8]-[12].

**Table 1:** **Technology, platforms and features of the currently available sequencing methods**

Sequencing technology	Platform	Mb/run^a^	Time/run	Read length (bp)	Limits	Applications
Sanger di-deoxy nucleotide sequencing	Capillary sequencers, for example, Life Technologies ABI3730	0.44	7 hours	650-800	Cost, need for high DNA amounts, cloning step	*De novo* sequencing
Pyrosequencing	Roche (454) GS-FLX	700	24 hours	700	Difficulty in disambiguating repeat regions, misincorporation of excess nucleotides	*De novo* sequencing
	Roche (454) GS Junior	35	4 hours	250		
Sequencing by synthesis	Illumina Genome Analyzer II	95 × 10^3^	14 days	2 × 150	Limited paired-end and targeted sequencing	Resequencing
	Illumina Hi Seq2500	6 × 10^5^	11 days	2 × 100		Resequencing
	Illumina MiSeq	15 × 10^3^	56 hours	2 × 300		*De novo* sequencing, resequencing
Ligation-based sequencing	Life Technologies SOLID 5500	32 × 10^3^	15 days	50 + 35	Specific sequence format, difficult sequence assembly	Resequencing
Semiconductor sequencing	Ion Torrent PGM	200	4 hours	200-400	Artificial insertions or deletions in mononucleotide repeats	Resequencing
	Ion Torrent Proton	2.5 × 10^3^	4 hours	100-200		
						Resequencing
SMRT technology	Pacific Biosciences PacBio RSII	0.5-1 × 10^3^	4 hours	10^3^-10^4^	Substitution errors	*De novo* sequencing and genome structure
Ionic current sensing	Oxford Nanopore Technologies	NA	No fixed run-time	10^4^-5 × 10^4^	NA	*De novo* sequencing
	MinION					

^a^Abbreviations: NA, data not available.

**Figure 1:**
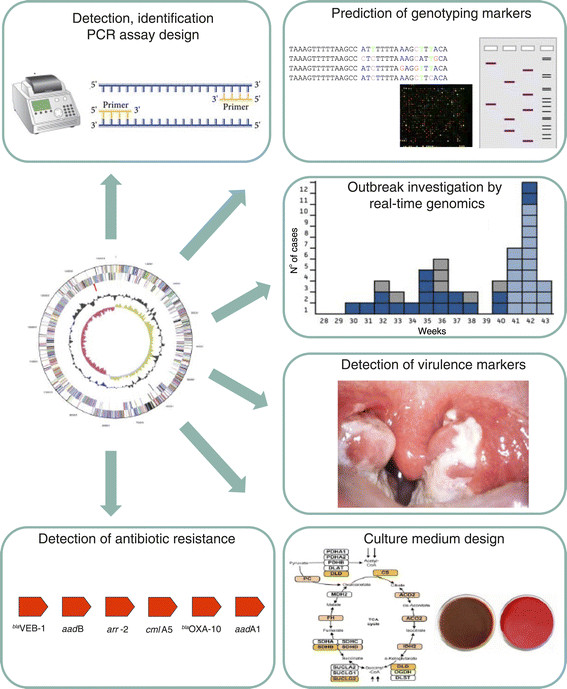
**Applications of bacterial genomics to the management of infectious diseases.** Genome sequence analysis has enabled the development of various clinical-microbiology tools for pathogen detection, identification or genotyping by identification of sequence fragments specific at distinct taxonomic levels (genus, species, strain, clone), for the detection of genes associated with antibiotic resistance or virulence and for the identification of deficient metabolisms to aid the development of optimized culture media. However, whole-genome sequencing, by giving access to the full genetic repertoire of an isolate, has demonstrated an undisputed discriminatory power for deciphering outbreaks of infectious diseases.

Here, we review the most relevant applications of genomics to the fields of molecular detection, identification and genotyping of infectious-disease agents, detection of virulence and antibiotic-resistance markers, design of culture media and investigation of outbreaks (Table 2; Figure 1), including those that are already available in clinical microbiology laboratories, and we offer our thoughts on how genomics might change clinical microbiology in the future.

**Table 2:** **Current applications of high-throughput genome sequencing in clinical microbiology**

Objective	Methods	Applications^a^	Examples [references]
Pathogen detection	Identification of target fragments and PCR primer design		*Mycobacterium paratuberculosis*[15]
			*Streptococcus pyogenes*[17]
	Syndrome-based detection	RT-PCR	Febrile illness [20]
		Multiplex RT-PCR	Tuberculosis [8]
		Microarray	Pneumonia [22]
	Highly sensitive molecular detection	PCR targeting multi-copy targets	Whipple’s disease [23]
		Suicide PCR	Rickettsioses [24]
Genotyping	DNA banding methods	Pulsed-field gel electrophoresis, PCR-RFLP	*Yersinia pestis*[9]
	Presence/absence of genes	RT-PCR	*Acinetobacter baumannii*[29]
	Presence/absence of repeats	MLVA	*Mycobacterium tuberculosis*[32]
	Presence/absence of point mutations	SNP detection	*Bacillus anthracis*[35]
	Whole-genome typing	Microarray	*Escherichia coli*[44]
		Genome sequencing	*Staphylococcus aureus*[61]
	Multiple gene sequencing	MLST	*Escherichia coli*[49]
	Multiple non-coding fragment sequencing	MST	*Rickettsia* species [56]
Detection of virulence markers	Comparison of virulent/avirulent strains		*Yersinia pestis*[10]
	Identification of lateral gene transfer		*Salmonella Enterica*[73]
	Search for known virulence factors in public databases		*Campylobacter* species [77]
Detection of antibiotic resistance	Comparison of resistant/susceptible strains		*Streptococcus pneumoniae*[96]
	Detection of antibiotic resistance markers in clinical isolates and specimens	RT-PCR	*Staphylococcus aureus*[100]
Culture medium design	Detection of defective metabolic pathways	Design of specific culture media	*Tropheryma whipplei*[12]
Outbreak investigation	Genome comparison	WGS	*Escherichia coli*[118]

^a^Abbreviations: bp, base pair; Mb, megabase; MLST, multi-locus sequence typing; MLVA, multiple variable number tandem repeat analysis; MST, multi-spacer typing; RFLP, restriction fragment length polymorphism; RT-PCR, real-time polymerase chain reaction; SNP, single nucleotide polymorphism; WGS, whole-genome sequencing.

## Detection of pathogens in clinical specimens

Rapid detection and identification of infectious agents in clinical specimens are mandatory in order to implement appropriate therapeutic measures. Therefore, an ideal detection assay should both be sensitive, specific and rapid to maximize the chances of patient recovery and be able to minimize the occurrence of clinical complications.

Since its development in 1983, PCR remained the most widely used molecular method in clinical microbiology, notably for detection of microorganisms in clinical specimens, until 1996 when real-time PCR (RT-PCR) was developed. In contrast to long-established culture-based diagnostic methods, PCR enabled identification of microorganisms regardless of their culturability and was, therefore, especially valuable in patients who had received antibiotics before sampling or those infected by fastidious microorganisms - that is, microorganisms that do not grow in the usual culture conditions [3]. However, early PCR assays were empirically designed and often targeted a gene common to all bacteria, thus allowing the detection of any species (for example, the rRNA operon or the *groEL* gene). Although these broad-range PCR assays enabled the discovery of many human pathogens [13], they suffered from various drawbacks, in particular a lack of sensitivity, specificity and discriminatory power among bacterial species [14]. By contrast, RT-PCR, targeting shorter fragments and using a fluorescent probe, greatly improved the speed, sensitivity and specificity of detection, in particular when coupled to the rational selection of PCR targets in genomic sequences according to the experimental objective and the degree of specificity required (genus-, species-, subspecies-, strain- or gene-specific) [15]-[17]. As the genomes from more than 37,000 bacterial strains are currently available, including those of all major human pathogens, it is now possible for clinical microbiologists to design specific PCR assays according to their needs by using the available tools. As examples, Marshall developed ‘PerlPrimer’, a software enabling the design of target-specific PCR or RT-PCR primers [15], Pritchard and colleagues proposed an alignment-free method for designing strain-specific primers for *Escherichia coli* O104:H4 [18], and Hung and associates designed a stepwise computational approach mixing several publicly available softwares to identify species-specific signatures in whole-genome sequences [17]. Using *Streptococcus pyogenes* as a model, Hung and colleagues designed and tested the validity of 15-signature-derived primer sets, including nine that were highly species-specific *in vitro*[17]. In addition, RT-PCR made possible the development of syndrome-driven molecular diagnosis in which assays detecting the most common etiological agents of a given syndrome are tested concomitantly [19]. In a recent study, Sokhna and colleagues described the use of a syndrome-driven strategy for the point-of-care diagnosis of febrile illness [20]. This type of diagnostic method has the advantage of testing, in a short time and a limited number of specimens, the most common causative agents of a given syndrome and can be especially valuable, for example, in the diagnosis of meningitis, pneumonia, endocarditis, pericarditis or sexually transmitted diseases. Thus, it enables a more efficient management of patients by enabling an earlier commencement of appropriate antibiotic therapy. Furthermore, genomics has also allowed the design of multiplex PCR assays enabling simultaneous detection and discrimination of various microorganisms, as has been the case for members of the *Mycobacterium tuberculosis* complex and *Mycobacterium canettii*[8]. This is also true for microarrays, some of which can enable the detection and identification of more than 2,000 viral and 900 bacterial species at once [21]. Nsofor recently reviewed the applications of microarrays to the syndrome-based diagnosis of infectious diseases, some of which, such as the ResPlex II Panel v2.0 (Qiagen, Hilden, Germany) and the FilmArray Respiratory Panel (BioMerieux, Marcy L’Etoile, France) for respiratory infections, are commercially available [22].

In addition to the development of highly specific PCR assays, the study of genomic sequences enabled the optimization of the sensitivity of detection, either by selecting a gene or fragment of noncoding DNA present as several copies in the genome [23] or by designing nested PCR assays targeting previously unused genomic fragments [24]. Fenollar and colleagues identified a seven-copy fragment in the genome from the bacterium *Tropheryma whipplei* and demonstrated that a RT-PCR assay targeting this repeated fragment was significantly more sensitive than assays targeting a single-copy fragment [23]. By contrast, Drancourt and colleagues developed a strategy named 'suicide PCR' that is based on nested-PCR assays targeting genome fragments that had never been used as PCR targets previously and that will be targeted only once with single-use primers [25]. These authors also demonstrated a higher sensitivity of their method over regular PCR. Targeting multicopy fragments was demonstrated to be highly sensitive for the detection of Q fever, Whipple’s disease, brucellosis, and infections caused by *Mycoplasma pneumoniae* or *Neisseria meningitidis*, whereas ‘suicide PCR’ was successful in detecting *Yersinia pestis* from dental specimens of ancient plague outbreaks and *Rickettsia* spp. in various arthropod-borne diseases [24],[25].

To date, several genome-based PCR tests have become commercially available. These include the LightCycler SeptiFast (Roche, Mannheim, Germany) and GeneXpert (Cepheid, Sunnyvale, CA, USA) systems that offer multiplexed detection of the various pathogens potentially involved in a given infectious syndrome. The latter system also enables simultaneous discrimination of *M. tuberculosis* complex species and detection of rifampicin resistance. Alternative assays are based on various detection methods for PCR products, as is the case for the ResPlex II Panel (Qiagen, Hilden, Germany) and Film Array (BioMerieux), in which PCR amplicons are hybridized to a microarray for the syndrome-based detection of pathogens, the GenoType MTBDR*plus* assay (Hain Lifescience, Nehren, Germany) that combines PCR and hybridization to a strip to detect antibiotic resistance in *M. tuberculosis*, and the PLEX-ID (Abbott, Abbott Park, IL, USA), in which broad-range and clade-specific PCR products are identified through using electro-spray ionization-mass spectrometry. The latter system enables screening human specimens for bacteria, viruses, fungi, protozoa and several antibiotic-resistance-associated genes [26].

However, although PCR and, more recently, RT-PCR have revolutionized the diagnosis of infectious diseases by reducing the time to diagnosis and increasing the detection sensitivity, several challenges remain, including the spectrum of detected agents, which is limited by the specificity of the assays used. However, thanks to their decreasing cost, the development of syndrome-based multiplex PCR assays or microarrays is likely to increase in the coming years. Alternatively, NGS, already known to be used for genotyping purposes in clinical microbiology, might also be increasingly used for clinical detection of pathogens, as was recently described for the diagnosis of a case of neuroleptospirosis [27].

## Genotyping

**Figure 2:**
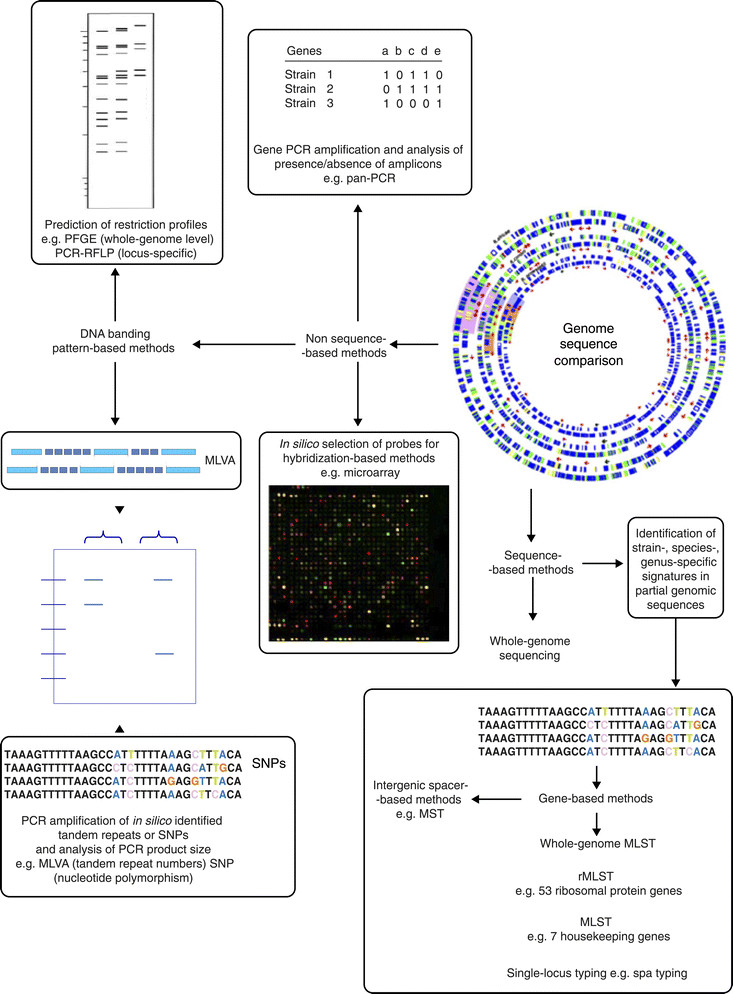
**Principles of genome-based genotyping methods.** By genomic comparison, investigators can identify specific sequence signatures that can be used in non-sequence-based methods (DNA banding-pattern-, PCR- or hybridization-based methods) or sequence-based methods (partial or complete genome sequencing). MLST, multi-locus sequence typing; MLVA, multiple locus variable number tandem repeat analysis; MST, muti-spacer sequence typing; PCR-RFLP, PCR-restriction fragment length polymorphism; PFGE, pulsed-field gel electrophoresis; RFLP, restriction fragment length polymorphism; SNP, single nucleotide polymorphism.

In situations when understanding the source and spread of microorganisms is crucial, as is the case for outbreaks caused by multidrug-resistant or hypervirulent bacteria and nosocomial or pandemic infections, a higher discriminatory power is needed to be able to trace pathogens at the strain level. Identifying bacteria at the strain level - or bacterial strain typing - is particularly important for epidemiological surveillance of infections. Strain typing also has applications in studying bacterial population dynamics. Over the past three decades, molecular typing (or molecular fingerprinting) methods have largely superseded phenotypic methods, including the morphology of colonies on various culture media, biochemical tests, serology, killer toxin susceptibility and pathogenicity, which exhibit insufficient discriminatory power, inability to quantify genetic relationships between isolates, limited reagent availability, poor intra- and inter-laboratory reproducibility and difficulties in comparing results obtained in different laboratories. In a similar fashion as described for PCR assay design, genomic sequences can be a source of genotyping targets. Molecular typing methods can be classified as non-sequence-based and sequence-based genotyping methods, depending on their design (Figure 2). Non-sequence-based genotyping methods include pulsed-field gel electrophoresis (PFGE), PCR-restriction fragment length polymorphism (PCR-RFLP), multiple-locus variable-number tandem-repeat analysis (MLVA), single-nucleotide polymorphisms (SNPs) and microarrays. Sequence-based genotyping methods include multilocus sequence typing (MLST), multispacer sequence typing (MST) and whole-genome sequence typing. The choice of genotyping method should be made according to the population structure of the investigated microorganism. This is particularly crucial for clonal bacteria, such as *M. tuberculosis* or *Bacillus anthracis*, for which structural genes are poorly polymorphic and PCR-RFLP or MLST are inadequate, whereas MLVA is able to discriminate among strains [28].

### Non-sequence-based genotyping methods

PFGE and PCR-RFLP have long been considered as 'gold standard' genotyping methods. These methods are DNA-banding-pattern-based methods that compare the electrophoretic profiles of restriction-enzyme-cut genomes or PCR-amplified genes from various strains. Initially, these methods relied on uncharacterized genomic differences or empirically selected target genes. By contrast, genome sequences, as was the case for *M. tuberculosis* or *Y. pestis*[9], can be used to rationally improve the sensitivity and specificity of PFGE or PCR-RFLP by enabling the ‘*in silico*’ prediction of the most appropriate restriction profiles of rare-cutter enzymes for a given bacterium.

In an alternative approach, Yang and colleagues have used genomics to design the ‘Pan-PCR’ software, dedicated to the identification of strain-specific PCR targets in genome sequences in a ‘presence/absence’ mode, that is, the amplification of a series of unrelated genes that were differentially present in the genomes from the studied strains [29]. As an example, in *Acinetobacter baumannii*, the presence or absence of six genetic loci, as determined by six locus-specific PCR assays, discriminated 29 tested strains [29]. Such a method is rapid, easy to perform and only requires a real-time thermal cycler, but it might not be adapted to species with highly conserved genomes such as *B. anthracis* in which the gene content does not vary among strains.

Another non-sequence-based genotyping method that benefited from the availability of genome sequences is MLVA. This method is based on the determination of the number and length of variable number of tandem repeats (VNTRs) present in a genome and is applicable to a variety of pathogens [30],[31]. Currently, MLVA is a reference genotyping method for many bacteria, such as *M. tuberculosis*[28],[32], and has also been used to investigate outbreaks of infections, as was demonstrated by Paranthaman and colleagues, who accurately identified the source of a multidrug-resistant *Salmonella enterica* serovar Typhimurium outbreak that occurred in England in 2011 [31]. MLVA is a rapid, easy-to-perform, affordable and reproducible genotyping method with high discriminatory power, but it has been demonstrated to be non-adaptable for some species, such as *Mycoplasma hyopneumoniae*, which lacks tandem repeats [33], and in long-term epidemiology for *Mycobacterium leprae* in which variations in the VNTR pattern were observed not only between isolates but also between specimens from the same patient [16].

The detection of single nucleotide polymorphisms (SNPs), another widely used typing method for bacteria, has also been improved through using genome sequences. This method, based on point-nucleotide changes between strains of a given species, has enabled the genotyping of several bacterial pathogens [9],[34]-[39], including *Coxiella burnetii*[40]. Using SNP genotyping, Huijsmans and colleagues identified five genotypes of *C. burnetii* that were involved in the large outbreak of Q fever that occurred in the Netherlands between 2007 and 2012 [40]. By comparison with other genotyping methods, SNP-based methods are rapid, sensitive, easy to perform and unambiguous in result interpretation. However, it should be noted that interpreting SNP genotyping data is highly dependent on the algorithm, the reference sequence and the sequencing platform used, which highlights a need for standardization of the methods used.

Genome-based DNA microarrays, an intermediate between non-sequence-based and sequence-based methods, contain probes specific for some or all genes present in a genome [41]. This method enables simultaneous strain comparisons at a whole-genome level. It can be automated and is a fast, sensitive and high-throughput genotyping tool [16],[42]. Genome-based DNA microarrays were developed to genotype a number of human pathogens, including *Escherichia coli*[43], for which Geue and colleagues were able to discriminate 446 Shiga-toxin-producing *E. coli*[44]. DNA microarrays can also be used to detect and identify microorganisms in complex floras [30],[45]. However, although highly discriminatory, microarray-based methods suffer from the major drawback that they cannot identify genetic fragments for which no probe is used.

### Sequence-based genotyping methods

By comparison with non-sequence-based methods, sequence-based genotyping has the major advantage of being highly reproducible because the sequence fragments on which it is based are stored in public databases. Sequence-based genotyping methods can rely on the selection of one or several genomic targets or on the whole genome sequence. Single-locus sequence-typing methods require the *in silico* identification of a highly variable gene, such as the coagulase- and protein-A-encoding genes that are the genomic targets of coa or spa typing, respectively, two broadly used tools for *Staphylococcus aureus*[46],[47].

MLST, developed in 1998, is one of the most frequently used sequence-based genotyping methods. It is based on the combination of genotypes obtained from several individual genes, usually housekeeping genes, for characterizing bacterial strains [48]. Genome-sequence-designed MLST assays have been useful for typing pathogens that have highly variable genomes among strains, such as *E. coli*, *N. meningitidis* or *S. aureus*[30],[49],[50], but they demonstrated limited discriminatory power among those bacteria with highly conserved genomes such as *B. anthracis*[30]. In 2012, rMLST, based on a combination of 53 ribosomal protein subunits, was demonstrated to discriminate strains within the genus *Neisseria*[51]*.* However, whole-genome MLST, incorporating more than 500 loci, was able to identify bacteria at the clone level [52]. This method is especially valuable when implemented with the BIGSdb platform that enables standardization of data [53]. In a similar fashion, multi-spacer typing (MST), based on the assumption that intergenic spacers are more variable than genes owing to a lower selection pressure, combines sequences from the most variable intergenic spacers between aligned genomes of bacterial strains instead of genes [54]. First developed for *Y. pestis*[54], MST has also been efficient at typing strains from various other bacteria, including *C. burnetii*[30],[55]-[57]. Glazunova and colleagues, by using a combination of 10 intergenic spacer sequences, were able to classify 159 *C. burnetii* isolates within 30 distinct genotypes [55]. MST was demonstrated to be more discriminatory than MLST for *R. conorii* strains [56].

However, bacterial whole-genome sequencing (WGS) using NGS, by giving access to the whole genetic content of a strain, is the ultimate discriminatory sequence-based genotyping method and has already demonstrated its usefulness for epidemiological investigations, showing the rapid global transmission of infectious diseases [38],[58],[59] (Table 3). WGS was used to compare 86 human *M. tuberculosis* isolates from a German outbreak and has demonstrated its superiority over other genotyping methods for tracing and investigating micro-epidemics [60],[61]. In 2010, WGS was used to study 63 strains of methicillin-resistant *Staphylococcus aureus* (MRSA) from various countries and enabled reconstruction of intercontinental transmissions over four decades as well as the potential transmission within a hospital environment [38]. WGS was also used to investigate the cholera outbreak in Haiti that occurred in 2010 [58],[59], revealing that Haitian strains were closely related to strains from Nepal. These pioneering studies demonstrated the potential of WGS for retrospective genotyping. The major challenge is to make WGS a genotyping tool during the course of outbreaks, and for this it will be necessary to facilitate access to sequencing platforms.

**Table 3:** **Examples of infectious disease outbreaks for which next-generation sequencing has been used**

Causative agent	Date of outbreak^a^	Country	Setting	NGS platform	Impact on disease control and/or findings	Reference
Multi-drug resistant *Acinetobacter baumannii*	2009	UK	Hospital	Roche GS-FLX	Proof of patient-to-patient transmission	[114]
*Bordetella pertussis*	2012	USA	Community	PacBio RS	Identification of several concomitant clones	[131]
*Clostridium difficile*	2007-2011	UK	Hospital and community	Illumina	Only one-third of cases were acquired from symptomatic patients	[132]
Carbapenem-resistant *Enterobacter cloacae*	2008-2009	UK	Hospital	Illumina MiSeq	Retrospective identification of two distinct strains	[133]
Vancomycin-resistant *Enterococcus faecium*	NA	UK	Hospital	Illumina MiSeq	Retrospective identification of the clonality of the causative strain	[133]
*Escherichia coli* O104:H4	2011	Germany	Community	Ion Torrent PGM, PacBio RS	Identification of the source of infection	[134],[135]
*Francisella tularensis holarctica*	2010	Sweden	Community	Ion Torrent PGM, PacBio RS	Retrospective identification of several clones	[136]
Carbapenemase-producing *Klebsiella pneumoniae*	2012	Nepal	Hospital	PacBio RS, Illumina HiSeq	Identification of a clone responsible for three distinct outbreaks	[137]
*Legionella pneumophila*	2012	Canada	Community	Illumina MiSeq	Identification of the source of infection	[138]
*Listeria monocytogenes*	2008	Canada	Community	Roche GS-FLX	Retrospective identification of three clones responsible for a nationwide outbreak	[139]
*Mycobacterium abscessus*	2007-2011	UK	Cystic fibrosis center	Illumina HiSeq	Proof of patient-to- patient transmission	[140]
*Mycobacterium tuberculosis*	2006-2008	Canada	Hospital	Illumina Genome Analyzer II	Retrospective identification of two concomitant outbreaks	[141]
*Mycobacterium tuberculosis*	2010	UK	Community	Illumina MiSeq	Identification and treatment of contact patients	[119]
*Neisseria meningitidis*	1997	UK	Hospital	Illumina Genome Analyzer II	Retrospective identification of the causative clone	[142]
*Salmonella Newport*	2011	Europe	Community	Illumina HiSeq	Confirmation of watermelons as source of international spread of a *Salmonella newport* clone	[143]
*Salmonella Enteritidis*	2010-2012	USA	Hospital	IonTorrent PGM	Retrospective and prospective identification of a single clone responsible for the outbreak	[144]
Methicillin-resistant *Staphylococcus aureus*	2009	USA	Pediatric hospital	Illumina MiSeq	Retrospective identification of the causative clone and its resistome	[61]
Methicillin-resistant *Staphylococcus aureus*	2011	UK	Hospital	Illumina HiSeq	Identification of carriage by a staff member	[145]
*Staphylococcus aureus*	2011	USA	Hospital	Illumina HiSeq	Proof of absence of patient-to-patient transmission	[146]
*Vibrio cholerae*	2010	Haiti	Community	PacBio RS	Identification of the source of the causative clone	[59]
*Vibrio cholerae*	2012	Guinea	Community	Illumina MiSeq	Identification of the source of the causative clone	[147]

^a^Abbreviations: NA, data not available.

## Detection of virulence factors

In addition to identifying bacteria at various taxonomic levels, WGS offers the opportunity to detect various genetic markers, such as virulence factors or antibiotic resistance-associated genes. Identifying and characterizing the virulence factors of pathogens are crucial for understanding the pathogenesis of the diseases that they cause and for developing dedicated molecular tools to detect specific virulence markers. However, among the currently known virulence markers, only toxins are important for optimizing the management of patients, as these agents are able to cause hospital outbreaks of severe infections with high mortality rates, such as the hypervirulent ribotype O27 *Clostridium difficile*[62], or because the administration of antibiotics can have a significant impact on the outcome. This is notably the case for *S. aureus*, in which the secretion of the Panton-Valentine leukocidin is induced by oxacillin or depressed by clindamycin [63],[64], for the Shiga-toxin production in *E. coli* that is stimulated by β-lactams, sulfonamides and fluoroquinolones [65], and for *Rickettsia conorii*, in which fluoroquinolones upregulate a toxin-antitoxin module [66]. Therefore, determining the toxinic repertoire of strains of selected bacterial species can be crucial for effective clinical management.

Genomics has played an important role in the identification of virulence factors in bacteria. Three main strategies are used to identify virulence-factor-encoding genes in genomes [67]: first, comparison of genomes from strains or species exhibiting diverse degrees of virulence; second, identification of laterally transferred genomic islands, assuming that virulence genes are often acquired by this mechanism [67]; and, third, running the genome against databases of known virulence markers. The first approach was used in studies between *Y. pestis*, the causative agent of plague, and the less-virulent but closely related species *Y. pseudotuberculosis*[10], between a pathogenic strain of *E. coli* O157:H7 and a non-pathogenic laboratory strain of *E. coli* K-12 [68],[69], between a highly virulent *Staphylococcus epidermidis* causing community-acquired endocarditis and commensal strains [70], and between *Klebsiella pneumoniae* strains [71]. The second strategy enabled the identification of pathogenicity islands in various species [72]-[75], such as *E. coli* or *S. aureus*. The third method enabled identification of virulence genes in a variety of species [76]-[87], notably *Listeria monocytogenes* and *M. tuberculosis*. All three strategies are complementary but cannot replace functional studies for confirmation of the real role of the identified virulence factors in pathogenesis.

Paradoxically, genomic comparisons have also questioned the paradigm of virulence by gene acquisition. In many genera, genome reduction, rather than acquisition of additional genetic material, can be associated with increased virulence, as many of the most virulent bacterial pathogens have smaller genomes than closely related species [88]. The comparison of rickettsial genomes showed that *Rickettsia prowazekii*, the agent of epidemic typhus, the deadliest rickettsiosis, had the smallest genome in this genus (Figure 2) [89]. Similar findings were reported for *Mycobacterium ulcerans*[90]*.* In addition, the presence of ‘non-virulence’ genes was described as discriminating *Shigella dysenteriae* from *E. coli* or *Y. pestis* from *Y. pseudotuberculosis*[88]. In *Y. pestis*, for example, the loss of the *rcsA* and *nghA* genes, which encode a repressor of biofilm synthesis and an inhibitor of biofilm formation, respectively, might have contributed to a more efficient flea-borne transmission [91]. Therefore, the pathogenic repertoire of a bacterium should not only take into account the presence or absence of virulence factors but also of ‘non-virulence’ genes.

However, it should be noted that the virulence of a bacterial strain might not systematically be predicted from its genome sequence, in particular when the identified virulence markers are not expressed. Indeed, Priest and colleagues could overcome this limitation by using systems biology to predict virulence in *S. aureus*[92]. Briefly, these authors not only considered the presence of virulence genes but also took into account the known regulatory networks of these genes.

## Detection of antibiotic resistance

Currently, antimicrobial resistance is a major public health concern worldwide, especially as some pathogenic multidrug-resistant bacteria are already resistant to all antibiotics in use in the clinic [93]. Detection of bacterial resistance determinants, and identification of new arrangements of known resistance genes, as well as new putative resistance markers can be achieved with WGS. This might help predict the resistance phenotype, set up enhanced in-hospital infection-control measures, adapt a specific therapy and enable the identification of resistance-causing genes or mutations that could be detected by PCR from clinical isolates or specimens and serve as targets for routine detection tools [94]. The strategies for identifying resistance markers are very similar to those aimed at identifying virulence genes [6]. However, as incomplete data link genotype to phenotype in terms of drug resistance, WGS genomic-based detection is particularly suited for antibiotics for which resistance-associated mutations or genes are known and notably for fastidious bacteria such as *M. tuberculosis*[95].

Genomic comparisons of phenotypically resistant and susceptible strains has enabled investigation of the resistome - that is, the repertoire of genetic markers associated with antibiotic resistance of multidrug-resistant strains of *Enterococcus faecium*[11] and *S. pneumoniae*[96]. Genome sequencing has also enabled identification of resistance mechanisms in fastidious bacteria that express few phenotypic characteristics, as was the case for *T. whipplei*, the causative agent of Whipple’s disease, that is resistant to fluoroquinolones owing to mutations in the *gyrA* and *parC* genes [97], *Rickettsia felis*, which expresses a β-lactamase activity that was first found in the genome [98], and *M. tuberculosis*, in which a putative rRNA methyltransferase might explain its resistance to macrolide antibiotic drugs [95].

Several PCR assays used in clinical practice derive from genomic sequences. The recent discovery of the *mecC* gene - a homolog of the *mecA* gene encoding methicillin resistance, responsible for false susceptibility testing results - in the genome of a methicillin-resistant *S. aureus*[99] elicited the design of specific PCR assays [100]. The spread of carbapenemase-producing enterobacteriaceae also prompted the sequencing of genomes from various MDR strains, including an NDM-1-producing *E. coli* strain [101] and a *bla*_KPC2_-producing *K. pneumoniae*[102], which in turn enabled the development of dedicated PCR assays [103]. Therefore, although many genome-based molecular tests facilitating the management of infections have already been developed to date, there is no doubt that WGS data will be used extensively in future assay design.

## Culturing unculturable pathogens

Despite the breakthrough of molecular methods, culture remains the cornerstone of routine microbiology as it provides insight into their ecology and pathogenicity. However, a majority of microorganisms in nature are not cultivable using standard techniques. Many fastidious bacteria grow poorly on commonly used media, and others are considered uncultivable on axenic media, possibly owing to deficient or partial metabolic pathways. Thus, genome sequences might enable identification of incomplete metabolic pathways [104] and the essential nutrients that a bacterium is unable to produce [105], which could then be incorporated into a specifically designed culture medium. *T. whipplei*, causing Whipple’s disease, was the first ‘unculturable’ human pathogen [106],[107] to benefit from such an *in silico* design of a culture medium. An axenic culture medium specifically designed to contain the nine amino acids that this bacterium was unable to synthesize enabled its axenic growth [12]. A similar approach was used for *Xyllela fastidiosa*[108], *Leptospirillum ferrodiazotrophum*[109] and *C. burnetii*[110]. Alternatively, genomics might help improve culture media, as was the case for *E. coli* and *M. pneumoniae*[111],[112]. However, this strategy might not be efficient for just any bacterium, as was the case for *M. leprae*. Despite the many important metabolic activities missing in the genome [113] of this bacterium, no specifically complemented axenic medium has enabled any growth to date. However, although it is important to improve culture methods for fastidious microorganisms, the investigation of unusual infections or outbreaks needs rapid and informative methods that may help influence the management of patients and course of the outbreaks. Such progress is now made possible by NGS.

## Real-time genomics for the diagnosis of infections or the investigation of outbreaks

The development of NGS bench-top sequencers such as the MiSeq (Illumina) and Ion Torrent Personal Genome Sequencer (PGM; Life Technologies) has made genome sequencing compatible with the routine clinical-microbiology workflow [6]. Such a strategy enables, within a few hours, exhaustive access to the genotype [39], virulence markers and antibiotic-resistance repertoire. Real-time genomics has notably been used to investigate several nosocomial [70],[114] or community-acquired infections [115]-[118] (Table 3). Sherry and colleagues used PGM sequencing of four MDR *E. coli* strains to confirm that the nosocomial outbreak that had occurred in a neonatal unit in Melbourne, Australia, had been caused by a unique clone and to characterize the resistance genes for this outbreak strain [118]. In Germany, Mellmann and colleagues compared the genomes from two *E. coli* O104:H4 strains from two hemolytic uremic syndrome outbreaks and concluded that the strains had diverged from a common ancestor and that NGS was suitable for the characterization of a pathogen in the early stages of an outbreak [115]. In both cases, genome sequences were obtained in a few days (five and three days, respectively). These findings demonstrated how rapid and precise genomic sequencing, although limited to a few clinical-microbiology laboratories currently, could transform patient management or improve hospital infection control in routine clinical practice.

Although only a few studies to date have described a turnaround time sufficiently short to enable WGS data to influence the course of outbreaks [119], the increasing number of teams using WGS for epidemiological purposes (Table 3) leaves little doubt as to the likelihood of its systematic use as a first-line tool to track and understand epidemics in the near future.

## How will next-generation sequencing change clinical microbiology?

NGS has the potential to change clinical microbiology in several ways. First, the increasing number of genome sequences will enable the development of new and improved pathogen-specific or syndrome-based single or multiplexed RT-PCR assays and will aid the refinement of DNA targets, primers and probes used in existing tests [120]. Second, the increase in speed, decreasing costs and discriminatory power of NGS make it an ideal tool for routine use in diagnostic microbiology laboratories. NGS has the potential to replace several existing tests performed on the same isolate, notably identification of antibiotic-resistance mechanisms, virulence determinants and genotype, in particular for microorganisms that are difficult to grow. As such, it is especially well suited for infection control. In addition, NGS without the need for culture, in particular single-cell sequencing, might be relevant for the routine characterization of unculturable bacteria. Third, NGS has proven its usefulness to decipher complex microbiotas in various metagenomic studies [121]. Recent studies have demonstrated its ability not only to discriminate among microorganisms present in human specimens, and thus possibly detect co-infections, but also uncover unexpected or new pathogens [122]-[124].

However, several challenges remain, the most important being a facilitated and rapid access of clinical microbiology laboratories to sequencing platforms, and a need for standardized and fully automated sequence interpretation that would ideally be independent of both the sequencing platform and the exact species of microorganism [125]-[127]. Also needed is the ability to translate the data into relevant information enabling microbiologists, clinicians and public-health epidemiologists to implement control measures in real-time and alter the course of outbreaks. This implies a constant update and curation of public databases as well as the development of systems-biology-based softwares that will enable prediction of virulence and antibiotic resistance from genome sequences.

## Conclusions and perspectives

The expansion of genomics, giving access to the genomes of virtually all human pathogens, has greatly changed our approach regarding management of infectious diseases by shedding light on their genetic diversity, pathogenesis, evolution, detection and treatment. With access to the full genetic content of microorganisms, rational selection of DNA fragments has enabled creation of a wide array of detection and typing methods as well as specialized tools for the identification of genes encoding factors affecting virulence or antibiotic resistance. In addition, NGS methods have reached a point, both in terms of cost and speed, where they might enter the routine microbiology laboratory and be used routinely for the rapid sequencing of microorganisms that exhibit unusual pathogenicity, are antibiotic-resistant or cause outbreaks. However, the major challenge in order to include genome sequencing in the routine workflow of the clinical-microbiology laboratory, in addition to a need for a multiplication of sequencing platforms, is a clear need for improved sequence analysis, both in terms of numbers and data handling of bioinformatic facilities, and storage capacity, as well as homogenized gene-function assignment.

It is likely that NGS, by permitting genome sequencing from single cells or single colonies, will also constitute a major step forward in the comprehension of bacterial genome dynamics [128]. This strategy has the advantage over other sequencing methods in that it is applicable to microorganisms that are unculturable and/or part of complex floras [129],[130]. However, single-cell genomics also currently suffers from several limitations, which include genome amplification biases, chimeric DNA rearrangements and a need for the improved *de novo* assembly of DNA sequences of previously non-sequenced microorganisms.

## Authors’ original submitted files for images

Below are the links to the authors’ original submitted files for images. Authors’ original file for figure 1 Authors’ original file for figure 2 Authors’ original file for figure 3

## Abbreviations

MLST: multi-locus sequence typing
MLVA: multiple variable number tandem repeat analysis
MRSA: methicillin-resistant *Staphylococcus aureus*
MST: multi-spacer typing
NGS: next-generation sequencing
PCR-RFLP: PCR-restriction fragment length polymorphism
PFGE: pulsed-field gel electrophoresis
RFLP: restriction fragment length polymorphism
RT-PCR: real-time polymerase chain reaction
SNP: single nucleotide polymorphism
VNTRs: variable number of tandem repeats
WGS: whole-genome sequencing
