# Supplementary figures and images for: Clinical detection and characterization of bacterial pathogens in the genomics era
Source: Genome Med. 2014 Nov 29;6:114. doi: 10.1186/s13073-014-0114-2 (PMC4295418; doi:10.1186/s13073-014-0114-2)

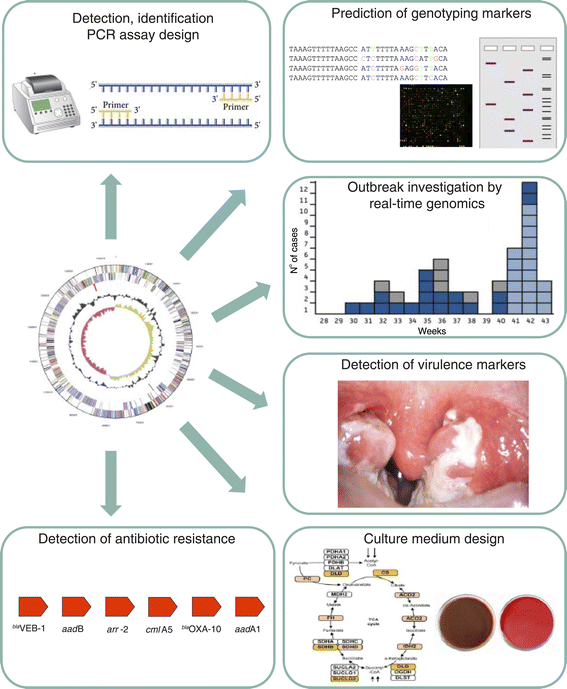

Supplement: Supplementary file 1 — Authors’ original file for figure 1 [file 13073_2014_114_MOESM1_ESM.gif]

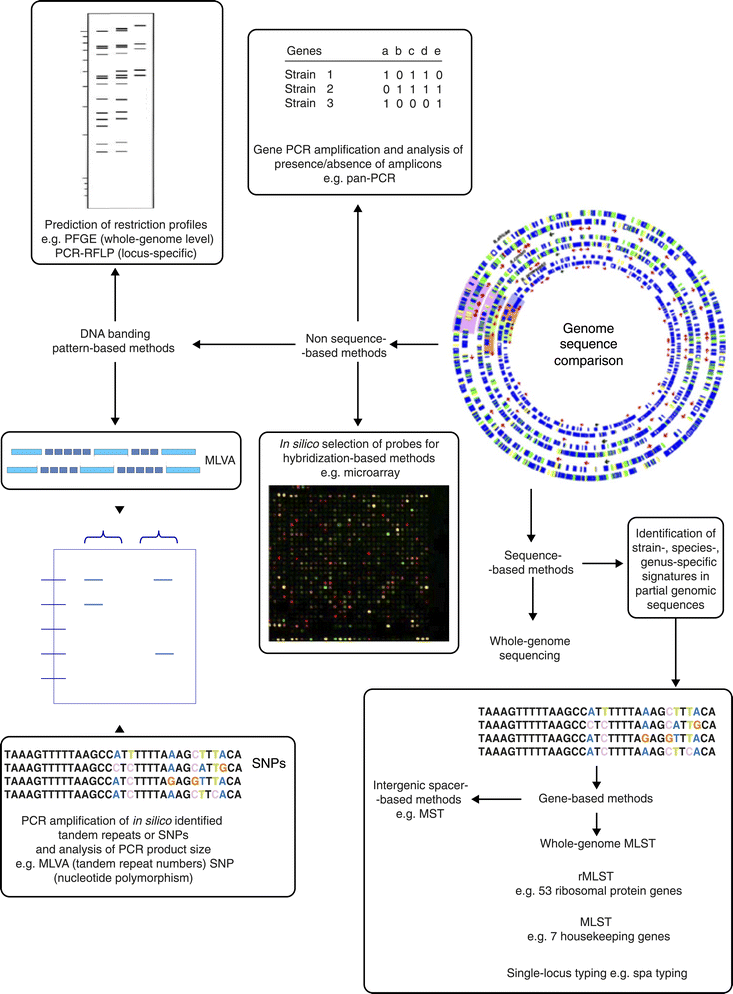

Supplement: Supplementary file 2 — Authors’ original file for figure 2 [file 13073_2014_114_MOESM2_ESM.gif]

## Slide 1
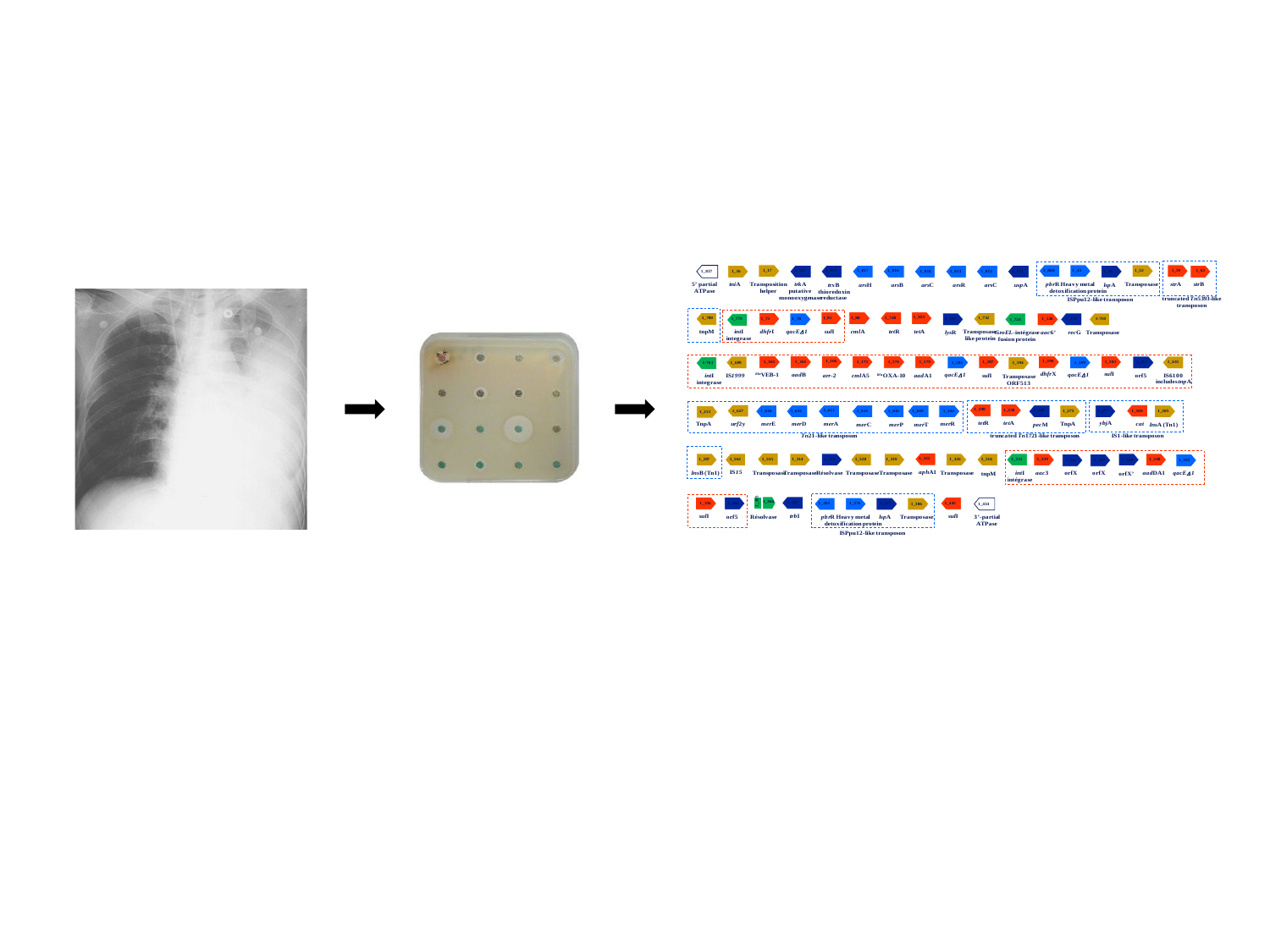

Supplement: Supplementary file 3 — Authors’ original file for figure 3 [file 13073_2014_114_MOESM3_ESM.pptx]
